# Suubi4StrongerFamilies: A study protocol for a clustered randomized clinical trial addressing child behavioral health by strengthening financial stability and parenting among families in Uganda

**DOI:** 10.3389/fpsyt.2022.949156

**Published:** 2022-11-23

**Authors:** Fred M. Ssewamala, Mary M. McKay, Ozge Sensoy Bahar, Proscovia Nabunya, Torsten Neilands, Joshua Kiyingi, Phionah Namatovu, Shenyang Guo, Noeline Nakasujja, Abel Mwebembezi

**Affiliations:** ^1^Brown School, Washington University in St. Louis, St. Louis, MO, United States; ^2^Vice Provost Office, Washington University in St. Louis, St. Louis, MO, United States; ^3^School of Medicine, University of California, San Francisco, San Francisco, LA, United States; ^4^International Center for Child Health and Development Field Office, Masaka, Uganda; ^5^College of Health Sciences, Makerere University, Kampala, Uganda; ^6^Reach the Youth Uganda, Kampala, Uganda

**Keywords:** children, Sub-Saharan Africa, child behavioral health, randomized clinical trial, disruptive behaviors, economic empowerment, family strengthening, combination interventions

## Abstract

**Background:**

Children in Sub-Saharan Africa are burdened by significant unmet mental health needs. Across the region, high rates of poverty, HIV/AIDS, food insecurity, stigma, and an inadequate health safety net system exacerbate serious child behavioral health needs and impede an effective response. Disruptive behavioral disorders are particularly concerning as they persist through adolescence and adulthood. Hence, addressing the context-specific social influences on child behavioral health is critical given that children in the region comprise more than half of the total regional population. Against this backdrop, this study protocol describes a randomized clinical trial that will examine the mechanisms by which economic empowerment and family strengthening interventions targeting social, familial, and context-specific drivers affect the mental health of children in Uganda.

**Methods:**

The study uses an experimental, longitudinal design across 30 cluster-randomized primary schools to compare single and combination intervention options; influences of economic empowerment and family strengthening on economic, perceptual, and functioning mediators; and context-specific moderators. The study will be conducted with 900 Ugandan children in mid-upper primary school (10–14 years). The three study conditions (*n* = 300 each) are: (1) economic empowerment only (EE only), (2) multiple family group-based family strengthening only (MFG-based FS only), and (3) combined EE + MFG-based FS. The interventions will be provided for 12 months; and assessments will occur at baseline, 12, 24, and 36 months.

**Conclusion:**

Children in Sub-Saharan Africa are burdened by significant unmet mental health needs, including disruptive behavior disorders that persist through adolescence and adulthood if left untreated. The proposed study will examine the mechanisms by which economic empowerment and family strengthening interventions targeting social, familial and context-specific drivers affect the mental health of children in mid-upper primary schools in Uganda. Findings from this study can inform group, community, and population approaches that are needed for scalable solutions to address the social drivers negatively impacting child behavioral health in low-resource settings, including in Sub-Saharan Africa.

**Clinical trial registration:**

[https://clinicaltrials.gov/], identifier [NCT053 68714].

## Introduction

Children in Sub-Saharan Africa (SSA) experience a significant rate of unmet mental health needs (1, 2). According to a recent systematic review, 1 in 7 children in the region struggle with a serious mental health issue (3). Across SSA, high rates of poverty, food insecurity, stigma, and an insufficient health safety net system further intensify serious child behavioral health needs and hinder an effective response. Moreover, higher rates of child behavioral challenges in the SSA region, a region heavily impacted by HIV/AIDS, have been found among children impacted by HIV/AIDS (1, 4–10).

Child disruptive behavior disorders (DBDs) are particularly concerning as they can persist through adolescence and adulthood, if not addressed in a timely manner. DBDs are also associated with poor physical health and interpersonal challenges in adulthood (4–7). Hence, the importance of addressing the context-specific social influences on child behavioral health, as well as the magnitude of the task cannot be overstated, given that children in SSA comprise more than half of the total regional population (1).

If children’s needs are to be met in SSA, then: (1) implementing interventions designed and tested in SSA, and which mobilize resources within existing child-focused institutions (families and schools) is critical (8–10); (2) combined interventions that simultaneously target SSA-specific influences on child behavioral health (family financial stability and culturally based parenting), and can be delivered in collaboration with child/family-serving community settings (schools and faith-based and financial institutions) are necessary (9); and (3) group, community, and population approaches to child behavioral health are needed to drive scalable solutions (9, 11, 12).

Savings-led economic empowerment interventions (13–25) have demonstrated to be efficacious in addressing the myriad of needs presented by children living in poverty- and AIDS-impacted communities in SSA, including improvement in mental health functioning (15, 17, 19, 26). Economic empowerment interventions directly target family financial stability and investment in the protection of children *via*: (1) incentivized matched Child Development Accounts (CDAs), (2) financial literacy training (FLT) and income-generating activities (IGAs) for families, and (3) mentorship. In addition, family strengthening interventions can positively impact CBH in SSA (27–29). Although scientists continue to disentangle the effects of culture and context on parenting and childhood DBDs across SSA, it is well recognized that the basic principles (e.g., behavioral supports, parent–child relationships, and involvement) underlying effective parenting practices are considered cross-culturally robust and play a critical role in child behavioral health (30–32). The multiple family group (MFG)-based family strengthening intervention to be tested in this study has shown to be efficacious in reducing oppositional defiant disorder symptoms and impaired functioning relative to usual care at 16 weeks (post-intervention) (33). The results also showed improved familial and peer relationships as well as better behavioral outcomes among children.

Against this backdrop, guided by Social Action (34), Asset (35, 36), and Family Systems (37) theories, the proposed study examines the mechanisms by which economic empowerment and family strengthening interventions targeting social, familial and_context-specific drivers affect the CBH of Ugandan children in mid-upper primary school (10–14 years). Specifically, the study uses an experimental, longitudinal design with three active study conditions across 30 cluster-randomized primary schools to compare single and combination intervention options; influences of economic empowerment and family strengthening interventions on economic, perceptual, and functioning mediators; and context-specific moderators. The study has the following specific aims:

Aim 1: Examine the impact of economic empowerment (EE only), multiple family group-based family strengthening (MFG-based FS only), and combined EE + MFG-based FS on children’s DBD symptoms and behavioral functioning;

Aim 2: Test the influence of EE only, MFG-based FS only, and combined EE + MFG-based FS on family financial stability (e.g., food and housing stability, material assets, and savings), parenting and protective family processes (e.g., family organization, caregiver/child interaction, cohesion, and support) and perceptions related to help seeking (e.g., stigma) on child behavioral health and functioning; and assess whether these change mechanism mediate intervention effects on DBD symptoms and behavioral functioning, and explore moderation by context-specific moderators of intervention effects;

Aim 3: Qualitatively examine participants’ experiences with each intervention arm.

## Background

A recent systematic review estimated that 1 in 7 children in SSA may struggle with a serious mental health issue (3). The WHO estimates prevalence rates may be even higher (20%) (38). In Uganda, 12–29% of children presented mental health symptoms when screened in primary care clinics (39, 40). Similarly, Nalunga (41) found that 1 in 5 Ugandan adolescents experienced a serious mental health challenge. In SSA, high rates of poverty, housing and food instability, effects of HIV and other health threats, and an inadequate health system exacerbate the prevalence of mental health needs and impede an adequate response.

Youth disruptive behavioral disorders (DBDs), including in SSA, are a particularly serious concern as they persist through adolescence and adulthood (4–7). The prevalence of DBDs in LMICs, including six SSA countries varies from 12 to 33% (3, 42–46). In a recent study with 2,434 school-going children in southwest Uganda screened for disruptive behaviors, 6% scored positive on oppositional defiant disorder and 2% scored positive on conduct disorder. In addition, 9.61% were reported to have elevated symptoms of oppositional defiant disorder (47). Children orphaned by AIDS or living with an HIV-infected parent were found to exhibit either an emerging or clinically serious DBD (13, 48–54). These prevalence rates translate into staggering numbers of children in need with child-serving systems not equipped to meet their needs (1, 4). For instance, in Uganda, there are only five child and adolescent psychiatrists, who are all located in urban tertiary care centers (55).

Disruptive behavior disorders are chronic, impairing, and costly mental health problems. When untreated, they can put youth at increased risk for future school drop-out, social impairment, substance use, delinquency, incarceration, criminal behaviors, unemployment, and premature death (4–7, 56–62).

### Risk factors for disruptive behavior disorders

Risk factors for an increased incidence of childhood DBDs include poverty, harsh parenting, poor caregiver–child relationships, large family size, stress, and the death of one or both parents (3, 31, 42, 43, 46). Given the negative consequences associated with childhood DBDs, it is critical that effective and scalable solutions that recognize the challenges facing most SSA countries are discovered. Specifically in Uganda, children constitute about half (56%) of the total population (compared to 20% in the US) (63) and simultaneously experience multiple physical, mental health, and academic challenges (55, 63). Ugandan children reside in communities that experience high rates of chronic poverty (38%), domestic violence (30%), physical violence toward children (80%), depression (33–39%), malaria (70–80%), and HIV/AIDS (6%) (4, 55, 64–67), and with a high number of orphans (4, 63). In addition, in order to effectively improve the mental health of the child population in SSA, one must address mental health stigma (68, 69), skepticism toward professional mental health support (2, 70–72), the large number of youth orphaned by HIV and other health epidemics, and limited economic opportunities (15, 19, 25, 73–80). Thus, culturally and contextually adapted family strengthening interventions that bolster SSA-specific family processes and parenting specific to SSA are needed to effectively address childhood DBDs. The group-delivered family strengthening interventions are specifically designed to target DBDs for youth whose families struggle with poverty and stress in the US and in SSA (27, 28, 81–83).

### Family strengthening interventions targeting disruptive behavior disorders

Positive behavioral supports, effective behavioral management, caregiver–child relationship, and caregiver involvement in a child’s life are critical to healthy child development regardless of families’ cultural or ethnic background. Several DBD-focused evidence-based practices (EBPs) are designed to enhance parenting skills (84–87) using behavioral practice, modeling, coaching, goal setting, family communication, and building on family strengths (2, 88–90).

Guided by Social Action (34) and Family Systems (37) theories, the family strengthening intervention recognizes the link between protective parenting practices and family processes, as well as contextual circumstances and supports in order to address childhood DBDs. Kazdin and Whitley (91) described how specific family factors associated with poverty (e.g., stress) may undermine parenting (e.g., family organization, discipline practices, family connectedness, support, and communication) and contribute to serious child behavior problems (15, 25, 80, 92–94). In collaboration with parents and service providers in the US, a MFG delivered family strengthening intervention was developed and guided by a protocol that encouraged transparency of the evidence base for families and provided an “easy to remember” means of organizing existing science for lay facilitators. Specifically, 4 broad conceptual categories are targeted at the family level for family strengthening (4Rs): Rules, Responsibility, Relationships, and Respectful communication. Stress and Social (2Ss) support were added given that these are factors that impact service engagement and outcomes among children raised within poverty-impacted families (39, 84, 92, 95–99). The 4Rs and 2Ss Family Strengthening Program is listed on the National Registry of Evidence-based Practices (100).

These components are embedded within the family strengthening intervention to be tested in the current study. Referred to as the 4Rs and 2Ss Family Strengthening program in the US, the AmaQhawe Family Program in South Africa (Strong Family in Zulu), and the Amaka Amasanyufu program in Uganda (Happy Families in Luganda), this intervention has been tested first in developed countries and in similar high poverty and high-stress communities in SSA – one of the few that is supported by data from SSA (27, 101). Specifically in Uganda, where the intervention was tested with children experiencing behavioral challenges and their families in a region heavily impacted by poverty and HIV, the results showed that the intervention was efficacious in reducing oppositional defiant disorder symptoms and impaired functioning relative to usual care at 16 weeks (post-intervention) (33). The results also showed improved familial and peer relationships as well as better behavioral outcomes among children.

### Economic empowerment interventions

Given the persistent threats associated with poverty, without attention to family economic stability, even with family strengthening interventions, families may not be able to fulfill their mental health promoting roles for children. Poverty affects families’ ability to physically care for children as well as family stability, functioning, and psychosocial wellbeing, thus constituting an important influence on child mental health. Studies have shown that family economic stability influences the quality of family relationships, where poverty negatively impacts caregiver–child communication and involvement (76, 102–104). In fact, the lack of material resources across contexts has been shown to undermine parents’ ability to foster children’s emotional and behavioral wellbeing by creating tense day-to-day interactions and compromising adult caregivers’ ability to positively direct children’s behavior (74, 105–111).

Studies have documented that the stresses and strains associated with poverty also can lower the connectedness between a child and his/her primary caregiver, which then predicts childhood mental health and functioning across domains (74, 105–111). Families who spend disproportionate time on material acquisition and survival have less frequent parent/child communication, thus driving poorer child psychological adjustment (74, 105–111). Moreover, families play a critical role in child development (including adolescence) and relatedly in any potential intervention designed to address the needs of young people (including the age group targeted by the proposed Suubi4StrongerFamilies intervention) (112). Indeed, the connectedness between a child and his/her primary caregiver can predict mental health functioning and overall child adjustment (113–115).

The parental role can provide an important protective factor for children who see themselves as connected to their families, and are thus less likely to suffer from mental disorders (115–121). Thus, particularly in resource-limited settings, providing families with economic opportunities can either maximize the benefits of family strengthening interventions or potentially eliminate the need for family strengthening entirely as economic empowerment diminishes the burden on families to rear children and support their behavioral success.

Economic empowerment interventions are guided by Asset theory (35, 122) according to which asset ownership can lead to many benefits, including expectations for more resources in the future, optimistic thinking, feelings of safety and security (123), and future planning (124). Asset building refers to efforts that allow people with limited economic opportunities to acquire and accumulate long-term assets (125). It is viewed as a critical strategy to reduce poverty, positively impact attitudes and behaviors, and improve psychosocial functioning (36, 125–127). Asset theory is consistent with other behavioral and psychosocial theories, including Bandura’s Social Cognitive Theory (128) and the Theory of Reasoned Action (129–134).

The economic empowerment intervention proposed in the current study draws on now robust evidence of the positive impact of savings-led economic empowerment approaches to improving family financial stability. Although similar to conditional cash transfers, which have become popular in the social development field and seek to enable families to meet their basic needs while incentivizing child behavioral protection (135–139), matched savings accounts proposed in this study go beyond that by underscoring long-term investment and promote life-long financial inclusion by developing savings habits and establishing partnerships among the participating families, financial institutions and the intervention program. For the proposed study, a CDA will be used, where savings are housed at a local bank and deposits made by the family are matched by the intervention to encourage savings. CDAs yield positive effects, including a greater sense of security, self-confidence, and future orientation (15, 16, 19, 74, 76, 77, 80, 140). CDAs also provide children and families with basic financial education, introduce them to formal financial institutions, and incentivize them to save small amounts by matching their deposits.

Given these complex mechanisms and pathways through which any given intervention may impact the overall mental health and psychosocial functioning of young people in SSA, investments in combination multi-level interventions are critical to providing an interdisciplinary, multi-level response desperately needed to improve child behavioral health and functioning in a way that single interventions alone have not yet sustainably been able to do. Thus, the current study examines the mechanisms by which economic empowerment and family strengthening interventions targeting social, familial, and context-specific drivers affect child behavioral health.

## Methods

**FIGURE 1:**
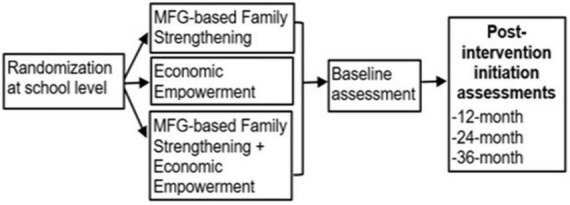
Study design.

This is a three-arm randomized control trial that will evaluate the mechanisms by which Economic Empowerment and Family Strengthening interventions targeting social, familial, and context-specific drivers affect child behavioral health (see Figure 1). More specifically, this study examines the direct impact of EE, FS, and combined EE + MFG-based FS on children’s DBD symptoms and behavioral functioning. In addition, the study examines the influence of EE, FS, and combined EE + MFG-based FS on family financial stability (e.g., food and housing stability, material assets, and savings), parenting, and protective family processes (e.g., family organization, caregiver/child interaction, discipline practices, cohesion, and support) and perceptions related to help-seeking (e.g., stigma) on child behavioral health and functioning. Finally, the study explores context-specific moderators of intervention effects (e.g., family circumstances, including deaths of adult caregivers, combined family structures, connection to community, and religious and cultural resources).

We expect to involve 900 youths aged 10–14 years in primary schools, grades 5 through 7, and their adult caregivers (900) in Uganda. The selected schools will be randomized to three study conditions: (1) EE only condition with a 1:2 saving incentive match—for educational purposes and microenterprise development (*n* = 300 participants; *n* = 10 schools); (2) MFG-based FS only condition (*n* = 300 participants; *n* = 10 schools; (3) combined EE + MFG-based FS condition (*n* = 300 participants; *n* = 10 schools). There will be four data points, these include baseline (pre-test), 12, 24, and 36 months post intervention.

### Study setting

According to the Ministry of Health’s most recent estimates, the HIV prevalence among adults (15–49 years) in Uganda is 5.4%, and 98,000 children (ages 0–14) are living with HIV (141). The study will be conducted with 900 youth aged 10–14 from 30 primary schools in five districts of Masaka, Rakai, Kyotera, Lwengo, and Kalungu in the Greater Masaka region, with 9.2% HIV prevalence rate (as opposed to 5.4% nationally) (141).

### Randomization

As in our prior studies (142, 143), stratified randomization of schools to conditions will be used, with schools stratified into four strata based on two variables: (1) student population size (medium size vs. large), and (2) geographical location (rural vs. urban), to ensure balance on those variables. The restricted randomization technique of Hayes and Moulton (142) will be used within the four strata to assure overall school balance across the three groups. Each of the 30 schools will be randomly assigned to one of the three study conditions and all selected students from a particular school will receive the same intervention (to reduce contamination). More specifically, 10 schools will be randomly assigned to receive an EE alone (*n* = 300 students), 10 to MFG-based FS intervention (*n* = 300 students), and the final 10 to the combined EE + MFG-based FS intervention condition (*n* = 300 students).

### Inclusion criteria

A total of 900 children will be recruited for the study. All eligible children within a school will be included and assigned to the same study arm. Youth will be eligible if they are: (1) in upper primary 5–7 (10–14 years) meeting criteria for oppositional defiant disorder or conduct disorder and willing to assent; (2) adult caregiver of the child willing to consent and available for research and intervention activities. A child will be excluded from participation in the study if they are; (1) unable to understand study procedures and participant rights as assessed during informed consent/assent process with the child or parent; (2) if the child or adult caregiver presents with emergency needs (e.g., hospitalization), needed care will be secured, rather than study participation. To avoid the stigma that surrounds having DBDs, no child will be excluded by virtue of their mental health screening status. If a child is not eligible for the study (does not evidence a DBD), the family will only be invited to attend the MFG meetings (all families in the school community will have the opportunity to attend to decrease stigma, but also enhance the functioning of all parents/families).

### Recruitment

Using the same recruitment procedures tested in our earlier studies (142, 143), we will rely on the schools and local district administration to identify participants and help with recruitment. We will make use of existing procedures at the beginning of the school academic terms during which each parent or caregiver comes to the school to meet with the administration to register their child (occurs within the first 2–4 weeks of the academic term). School administrators will give each child and parent a flyer that introduces the project and invites all the caregivers with an eligible child to contact the school for details. Community development officers and our implementing partner (RTY-Uganda) will also distribute flyers during their community visits to so that caregivers whose children meet the inclusion criteria but may not yet have reported back to school can also be informed.

Children and caregivers who indicate interest will be invited to come to the school in-person for a one-on-one information meeting with the research team, during which they will be given details of the project and will be informed, verbally and in writing, that study participation is voluntary. They will also be informed of the potential risks and benefits of participating in the program. The research team will obtain the informed consent of the primary caregiver and the assent of the child for all children. Parents will also be asked (following consent procedures) to complete screening tools for child DBDs. If the child meets the criteria for an emerging or clinically meaningful behavioral problem, they will be invited to enroll in the study and a baseline assessment will be completed prior to the start of the intervention.

### Retention and attrition

As in our previous studies (142, 143), we will ask participants to give their telephone number, and contact information for three people who will always know how to reach them, to help track their location only if we have lost contact. The research team will be in contact with all participants regularly during school roll calls to determine enrollment and attendance, as well as during the distribution and review of monthly bank statements for participants in the treatment conditions. This frequent contact will help to minimize loss to follow-up. We have effectively used these procedures in our previous research studies, resulting in very low attrition rates (7.3% over 5 years) (25, 75, 76). Based on these numbers, we conservatively expect attrition by end of follow-up to be no more than 20%.

### Ethics and informed consent

All study procedures were approved by the Washington University in St. Louis Review Board (IRB # 202202183) and by in-country local IRBs in Uganda: Uganda Virus Research Institute–UVRI (GC/127/901), and Uganda National Council of Science and Technology–UNCST (SS1205ES). Amendments to the study protocol will be submitted for approval to the above-mentioned IRBs. Participation in the *Suubi4StrongerFamilies study* will be voluntary. Written informed consent will be obtained from all participants. This will be done prior to assessment. In the consent form, it will clearly be stated that the participant can: withdraw from the study at any time, for any reason, with no explanation, and would not be penalized in any way; refuse to answer any questions at any time; review any materials and request that we erase any of their responses; make inquiries and address complaints to Secretary of the Ethics Committee at UVRI, UNCST, and Washington University in St. Louis. Participants will also be told of the potential risks and benefits of participating in the study. Each participant will receive a copy of the signed consent form.

### Study conditions

#### Economic empowerment intervention using matched savings accounts (child development account) intervention

Each child assigned to an EE group will receive a CDA held in a registered financial institution in Uganda. The child’s family members will be encouraged to contribute toward the CDA. The account will then be matched with money from the program. The maximum family contribution to be matched by the program will be an equivalent of US$10 per month per family. For children who save the maximum amount, they will have a total of $360 at the end of the intervention ($120 in savings plus $240 from the match: a 1:2 match). These amounts would be enough to pay for at least 3–4 years of a child’s upper primary educational expenses (e.g., examination registration fees, school uniforms, and any functional fees) in a public primary school. These are fees that are often prohibitive for poor school-going children to complete primary school and/or transition to secondary school to earn an ordinary level certificate that can be used to pursue advanced training or apprenticeship.

A monthly bank account statement will be generated for every child to note their accumulated savings. The statements are intended to act as “morale boosters” for the enrolled children. Also, during the intervention period, each child, with their primary caregiver as a co-signer, will have access to the money in their individual account (excluding the matching funds). In case of an emergency (for example, a family illness), participants can withdraw their own money—but not the matching funds. When the child turns 18, they will no longer need co-signers. As with our prior studies that used matching accounts (14, 19, 25, 74, 77, 143, 144), the matching funds will be kept in a separate account. To eliminate potential misuse of funds intended for education, payments from the matching fund will be made directly to the child’s school or wire transferred directly to the school’s bank account. The student will then contribute their portion of the total cost for the academic term. The same will apply to children who want to invest a portion in small business development: the check will be written to the vendors (or the wire transfer for the match will be sent directly to the vendor with the required particulars of the participant). This process is intended to eliminate the risk of family pressure on the children to withdraw money set aside for education. Participating students will be allowed to use up to 30% of their match funds to invest in a family-based income-generating activity (IGA), and the remaining 70% will be restricted only to fund the child’s education. Prior to accessing the match funds, participants will be required to complete 8 financial literacy management (FLM) workshops over a 12-month period. The in-country project coordinator will monitor the matching operations. This protocol has been successfully used in Uganda in our prior work (15, 16, 73, 76, 77, 80, 145).

The FLM workshops will consist of eight workshops that will: (a) introduce participants to the notion of asset-building; (b) cover asset-building strategies in detail, e.g., saving and investing in IGAs; and (c) cover specific topics related to saving, e.g., the importance of saving and how it can be done. As in our earlier studies, participants from the same school will be assigned to the same group during training, which will occur on weekends and school holidays.

#### The multiple family group-based family strengthening intervention

The MFG-based family strengthening approach is a hybrid of group and family interventions, rooted in several theories, including family systems theory, structural family theory, and social learning theory with elements of psychoeducation and social group work (146, 147). MFG has adopted the strengths of multiple therapeutic methods and theories to create an extremely flexible approach that has been applied to a variety of target populations struggling with a diverse range of issues (15, 16, 102, 104, 143, 145–148). MFG integrates components of existing EBPs found to successfully improve parental management and depression, mental health promoting family processes, and family strengthening (88). MFG is based on building support for parents and families by providing opportunities for parents and children to communicate in a safe setting with other families who have shared experiences (147). Advice and insight from other families is often seen as less threatening than feedback given by a therapist (147). In addition, MFG focuses on reducing stigma by normalizing shared experiences (147). Across country contexts, the family strengthening intervention is delivered in groups with multiple families present. More specifically, in the US, 6–8 families are included in each meeting. In South Africa and Uganda, 12–20 families are in attendance. At least 2 generations of a family (up to 20 families per group) are present in each session. Content and practice activities foster both within family and between family learning and interaction (149).

The MFG-based FS intervention acknowledges specific factors tied to poverty (e.g., stress) may undermine parenting (e.g., family organization, connectedness, support, and communication, and discipline practices) and contribute to poor mental health functioning (91) (see Table 1). The MFG-based FS approach will allow children and their families to share with other families in similar situations thus building hope by providing social support, normalization of similar experiences and struggles, and the sharing of effective solutions (150). The proposed study will utilize the MFG-based FS approach to specifically target family communication and supports both within and across families from the same community setting. In addition, MFG-based FS will aim to lower familial stress which may have negative impacts on children and overall family functioning. The MFG-based FS approach is an ideal way to build the protective factors of healthy parent–child relationships and communication while addressing familial, social and community stressors and barriers to children and adolescents’ mental health functioning.

**TABLE 1:** MFG-based family strengthening content.

MFG target (session #)	Empirically supported family skill	MFG goals
Rules (3 and 9)	Family organization; consistent discipline	Clarify rules, consequences, rewards
Responsibility (4 nd 10)	Inter-connectedness; expectancies	Clarify responsibilities, expectations, rewards
Relationships (5 and 11)	Family warmth; within family support	Schedule for positive family interaction
Respectful communication (6 and 12)	Family communication; family conflict	Listening/talking skills (parents/children)
Stress (7 and 13)	Parenting hassles and stress; life events	Identify stressors undermining family change
Social support (8 and 14)	Social isolation	Within family and external support plan

##### Description of family strengthening intervention protocol

The protocols have been designed to provide opportunities during each session to directly apply content to the realities of family life, emergent cultural and values perspectives, as well as tailor messages to the age of the child. We have built in redundancy for missed appointments and opportunities for reinforcement. We aim for families to attend at least 7–8 meetings, as findings suggest that this dose is needed to reduce child conduct problems and the majority of families reach this goal (83, 151).

##### Program delivery

At least six facilitators per school will be trained to facilitate the delivery of both the FS and EE + MFG-based FS interventions. Parent peers and teachers will be trained separately, based on study conditions. At the end of the program, facilitators will receive a certification in child mental health competency to be offered by the Ugandan Ministry of Health.

##### Training and supervision

Training will consist of up to 6 modules. Training focuses on childhood conduct difficulties, family-level factors that have been linked to child outcomes, strategies to enhance engagement and motivation, group facilitation skills, and processes specific to family strengthening. A knowledge and skills assessment test (KSAT), to assess mastery of the content, will be administered at the end of the training (152). Facilitators will receive 2 h of supervision per month, during the intervention period.

#### Combination intervention: Economic empowerment + multiple family group-based family strengthening

The combined arm will consist of: (a) an EE intervention that comprises a family monetary savings program using CDAs (detailed above), and (b) MFG-based family strengthening intervention focused on strengthening family relationships and mental health challenges (described above) frequently faced by children growing up in low-resourced communities characterized by poverty and disease (including HIV/AIDS). Combining these interventions recognizes that children’s mental health in poverty-impacted families is directly affected by the environment in which they live, with parental communication style and perpetuation of harmful social norms within a household (and community) potentially resulting in feelings of powerlessness and low self-worth. For those reasons, we are using the MFG model to build optimism, positive thinking as well as information on how to improve family processes as a mechanism to enhance mental health functioning.

### Data collection

All assessments (see Table 2) will take place at the participant’s home, ICHAD’s field offices in Masaka or satellite sites for study collaborators, with each lasting about 60–90 min. Although we expect most participants to be English-speaking (the instructional language in all Ugandan schools), assessments will be conducted in English or Luganda (local language) depending on participants’ English proficiency. All interviewers will be fluent in English and Luganda. Questions will be translated by a certified translator. Interviewers will receive a structured and intensive training conducted by the MPIs. All questions will be interviewer-administered. We will use unannounced school visits for school attendance using the same study protocols used during our Bridges and Suubi4Her studies (R01HD070727 and R01MH113486) (23, 153, 154). During school days, we will visit schools at an undisclosed time and day to do roll calls for students enrolled in the study once every month throughout the study period.

**TABLE 2:** Variables, instruments, reliability, and assessment time points.

Variable		Measurement	Reliability	Time point
**Demographics (respondent: child)**				
Age; orphan status; socioeconomic status; family stability		Socio-Demographic Questionnaire	n/a	B, 12, 24, 36

**Moderators (respondent: child)**				
Gender; orphanhood status; connections to community; utilization of religious resources				B, 12, 24, 36

**Potential mechanisms of change/mediators (respondent: child)**				
Mental health functioning	Adapted Child Depression Inventory (155)		0.65	B, 12, 24, 36
Post-traumatic stress disorder	The Child PTSD Index (156)		0.77	
Self-concept	Adapted Tennessee Self-Concept Scale (TSC-2) (25, 73, 75–78, 157)		0.81	B, 12, 24, 36
Family relations and cohesion; social support, family stability, duration of residence with family	Family Environment Scale/Family Assessment Scale (158–160)		0.54–0.87	B, 12, 24, 36
	Social Support Behaviors Scale (SS-B) (103, 161); MSPSS (74, 162)		0.69–0.84	
Stigma	Pediatric Self-Stigmatization Scale (163)		0.72–0.86	B, 12, 24, 36
Hopelessness and self-esteem	Beck Hopelessness Scale (155); Rosenberg Self-Esteem Scale (164)		0.77–0.88	B, 12, 24, 36
Food security/economic stability/assets	Household Food Insecurity Scale (165), asset ownership (166)		n/a	B, 12, 24, 36
Financial literacy and access to services	Financial literacy knowledge (167); RBA services (168)		0.80	B, 12, 24, 36
Savings (extracted from bank statements)	Monthly bank statements		n/a	B, 12, 24, 36
**Primary outcomes (respondent: caregiver)**				
Child disruptive behaviors	Disruptive Behavior Disorder Rating Scale (screening only),(47) Iowa Conners Scale (169), Impairment Scale (170)		0.73–0.79; 0.61–0.69	B, 12, 24, 36

#### Qualitative component (Aim 3)

Semi-structured interviews will be conducted at 12-month follow-up to explore the experiences of children and their caregivers with the intervention and key multi-level factors (individual, family, contextual, and programmatic) that may have impacted their participation. A stratified purposeful sampling strategy (171) will be used to randomly select participants from the highest (*n* = 5) and lowest (*n* = 5) quartiles on the main outcome (DBD) across the three study arms (*n* = 10 per arm; a total of 30 children and their caregivers) to be invited for in-depth interviews. This sample size will be sufficient for theoretical saturation (172–174). This method will ensure that participants with varying experiences within the same study arm are represented; allow us to identify patterns and variations in participants’ experiences and further understand reasons behind those performing higher and lower on the main outcome (DBDs). In addition, semi-structured interviews will be conducted with purposively sampled contact teachers given their direct engagement with participants and study team during study implementation (see C.3.11). We will identify schools with the lowest (*n* = 3 schools) and highest (*n* = 3 schools) intervention attendance rates in each of the three study arms (6 × 3 = 18 schools). Contact teachers (*n* = 18) in each of these schools will be invited to participate in semi-structured interviews exploring: (1) the perceived benefit of the intervention to the school; and (2) barriers and facilitators to implementation and uptake at the school level (e.g., resources, school readiness, and contextual relevance). Finally, we will invite one MFG facilitator from each of the 20 schools implementing MFG (*n* = 20 facilitators) to explore their experiences with the MFG-based FS intervention, including: barriers and facilitators to implementation and uptake at the individual (e.g., time, motivation, and competing demands), organizational level (e.g., resources and contextual relevance), and macro-level (e.g., cultural norms and stigma). All interviews will be conducted in English or Luganda based on participants’ preference. Each interview will last ∼60 min and will be audio-taped.

### Data analysis

#### Data quality assurance, initial analyses, and missing data

We will use MIS IDA Q (175) to check for data entry errors and missing values (175). Frequencies and measures of central tendency and variability will characterize the sample. We will address incomplete data with direct maximum likelihood (ML) and multiple imputation (MI) (175). Program code and results will be documented and archived to enable future access and dissemination.

#### Primary analyses for Aim 1

We hypothesize that: (1) H1a: Relative to participants in the MFG-based FS only group, participants in the combined EE + MFG-based FS will have lower mean levels of oppositional defiant disorders and functional impairment following the intervention; and (2) H1b: Relative to participants in the EE only group, participants in the combined EE + MFG-based FS group will have lower mean levels of oppositional defiant disorders and functional impairment following the intervention. To test hypotheses H1a–H1b, we will fit three-level linear mixed models (LMMs) to (1) the Iowa Connors measure of oppositional defiant disorder and (2) the Impairment Rating Scale (IRS). Each of these two models will include fixed effects for study arm, time, and their interaction. We will use random intercepts for School ID to account for clustering of persons within schools and include random intercepts, random slopes, and their covariance for person ID to account for clustering of repeated measurements within persons. To test H1a–H1b for each outcome we will perform two time-averaged comparisons of repeatedly measured observations across study arms to examine intervention effects over the duration of the post-intervention study period. Because two comparisons among the study arms will be evaluated per outcome, alpha (α) will be set at 0.05/2 = 0.025 for each of these planned comparisons.

#### Secondary exploratory analysis for Aim 1

While we do not have a formal hypothesis regarding the superiority of EE-only to MFG-based FS-only, when we fit the LMMs described above we will also explore whether EE only is superior to MFG-based FS only or vice versa. This exploratory comparison will be tested at α = 0.05.

#### Primary analyses for Aim 2: Evaluating the effects of the intervention on mechanisms of change

We hypothesize that: (1) H2a: Relative to participants in the MFG-based FS only group, participants in the combined EE + MFG-based FS group will have higher levels of family financial stability, parenting and protective family processes, and perceptions related to help seeking following the intervention; and (2) H2b: Relative to participants in the EE only group, participants in the combined EE + MFG-based FS group will have higher levels of family financial stability, parenting and protective family processes, and perceptions related to help seeking following the intervention. To test hypotheses H2a–H2b for mechanisms of change (e.g., asset ownership scale and food security) we will fit LMMs using the same fixed effects (study arm, time, and study arm-by-time interaction) and random effects for the school (random intercepts) and person levels (random intercepts, random slopes, and their covariance) as in the proposed H1 analyses described above. To test H2a–H2b we will perform two time-averaged comparisons of repeatedly measured observations of financial stability, parenting and protective family processes, and perceptions related to help seeking at α = 0.025 per comparison.

#### Primary analyses for Aim 2: Assessment of mediation of intervention effects

We will investigate whether family financial stability, parenting and protective family processes, and perceptions related to help-seeking constructs at 12 and 24 months mediate the relationship between intervention group assignment and DBD symptoms and behavioral functioning at 24 and 36 months, respectively. To maximize rigor, these analyses will be conducted using principles of structural equation modeling (SEM) and causal inference methods (176).

#### Secondary exploratory analyses: Moderation of intervention effects, including sex as a biological variable

All previously described inferential analyses will be repeated with models extended to include sex assigned at birth as a moderator to examine whether effects vary by participants’ sex. Additional moderators, including orphanhood status, connection to community, and utilization of religious and cultural resources, will be examined similarly. Alpha will be set to 0.05 for these exploratory analyses.

#### Secondary exploratory analyses for Aim 2: Comparing multiple family group-based family strengthening only to economic empowerment only

In the LMMs described above, we will also explore whether EE only is superior to FS only or vice versa at α = 0.05. We will also explore whether any observed EE only vs. MFG-based FS only difference is mediated and/or moderated by the potential mechanisms of change and moderators described above *via* SEM and causal mediation methods at α = 0.05.

#### Statistical power analysis

We used NCSS PASS (177) to estimate minimum detectable effect sizes for the LMMs proposed to test hypotheses H1a–H2b to fulfill specific Aims 1 and 2. For power analyses for the proposed LMMs we assumed power = 0.80, α = 0.05/3 = 0.025, and 3 repeated assessments from *N* = 720 participants from 30 schools based on conservatively assuming 20% attrition from our original sample of 900. Under these assumptions we computed the range of the minimum detectable standardized mean difference *d* as 0.24–0.29 for the LMM-based repeated measures analyses proposed to address Aims 1 and 2. For the mediation analyses proposed for specific Aim 2, we used NCSS PASS (177) to compute the minimum detectable standardized indirect effect *d* from a mediation analysis assuming power = 0.80, α = 0.05/2 = 0.025, and *N* = 720 participants following 20% attrition, yielding *d* = 0.33–0.37. Our proposed analyses have sufficient power to detect small to small-medium effects across a wide variety of possible analysis scenarios; these are similar to or smaller than effect sizes in the literature (178) and our studies (19, 104).

### Qualitative data analysis

Interviews will be transcribed verbatim and uploaded to QSR NVivo12 (179). Analytic induction techniques will be used for coding (156). Initially, 12 interview transcripts randomly selected across the study groups will be read multiple times and independently coded by the team using sensitizing concepts to identify emergent themes (open coding) (174). Broader themes will be broken down into smaller, more specific units until no further subcategory is necessary. Potential themes/subthemes include barriers and facilitators to the intervention participation at the individual-level (e.g., motivation, readiness to change, and time constraints); family level (e.g., competing demands and support); and program-level (e.g., content relevance; interaction with other program participants, site-specific concerns). For facilitator interviews, potential themes/subthemes include individual-level (competency, motivation, training, supervisory support, and readiness for change); and school-level (readiness, buy-in, and resources); and macro-level (e.g., cultural norms and stigma) facilitators and barriers. Similar themes related to school-level factors are expected in contact teacher interviews. Analytic memos will be written to further develop categories, themes/subthemes, and to integrate the ideas emerging from the data (174, 180). Codes and the inclusion/exclusion criteria for assigning codes (173) will be discussed as a team to create the final codebook. Each transcript will then be independently coded by two investigators to establish inter-coder reliability. A level of agreement ranging from 66 to 97% based on level of coding indicates good reliability (181). Disagreements will be resolved through team discussions. The secondary analysis will compare themes and categories within and across groups to identify similarities, differences, and relationships among findings. Member checking, peer debriefing, and audit trail will be used to ensure rigor (182).

### Data integration

Findings from qualitative and quantitative data analyses will be integrated at the interpretation and discussion stages (183). Conclusions and inferences will be synthesized for a more contextualized and thorough understanding of the participants’ experiences with the intervention. Data integration will serve two purposes: (1) *Complementarity* (184, 185); and (2) *Expansion* (184, 185). Qualitative findings will offer further explanations and context for findings from the quantitative analyses. The qualitative findings will: (1) expand on our understanding of attendance and participant satisfaction; and (2) inform our overall understanding of the impact of the intervention.

## Monitoring and responding to adverse events

The MPIs will train all study personnel in Uganda on identifying and identifying conditions that may jeopardize the welfare of study participants. Reporting of adverse events will occur according to a project protocol. For this study, safety and monitoring will be overseen by the project coordinator (based in Uganda), in-country co-investigator, and the MPIs. This group is expected to meet in person at least 2–3 times per year and will have weekly conference calls (using telephone, Zoom, and/or Skype). In the case of an adverse event, staff will inform the project coordinator immediately and the MPIs/in-country co-investigator within 24 h of the presence of a possible unanticipated adverse event. Any presence of a possible unanticipated adverse event will be immediately reported to the local research ethics committee in Uganda and Washington University Institutional Review. The IRBs will determine whether to stop the study protocol temporarily or provide suggestions and/or modifications to the study procedures. The research team will examine preliminary outcomes data on a quarterly basis to make sure there is no harmful impact on the study participants.

## Data management and integrity to protect confidentiality

To protect the integrity of the participants’ data, the following procedures will be followed. First, the data collected from the study participants will be used only for the purpose of research. All data will be kept confidential and will not be shared with anyone outside of the research team. Second, all participants will be assigned a code number, which will be used on all study-related documents and data collected from participants. Given the longitudinal nature of the study, we maintain lists of participants with links between identifying information and code numbers. Only the senior research team, comprising of the MPIs, in country co-investigator; project director (US)/coordinator (Uganda), and data manager will have access to these lists, which are kept in locked files. Other study personnel will have access on an as-needed basis to adequately perform their duties.

All study personnel will complete CITI Human Subjects Training, as well as training on data safety, participant confidentiality, limits of confidentiality, and proper administration of the study protocol. Hard copies of participants’ data will be stored in locked cabinets to which only the senior research team will have access. Only this group will have access to data entered into password protected computer files. All data use requests, will be reviewed by the MPIs. Only anonymized data, without identifying information will be shared. Study participants are notified of the above procedures in the informed consent/assent forms.

Participants are also informed of the limits of confidentiality, including mandatory reporting for child abuse and neglect under the Uganda law. Interviewers will receive training on the Ugandan laws regarding child abuse and/or neglect. Interviewers who suspect child abuse and/or neglect will be required to follow a specific reporting protocol intended to protect the safety of children, and at the same time reduce the risk that erroneous reports are made.

## Discussion

Children in SSA are burdened by significant unmet mental health needs, including DBDs that persist through adolescence and adulthood if left untreated. The Suubi4StrongerFamilies study will examine the mechanisms by which economic empowerment and family strengthening interventions targeting social, familial, and context-specific drivers affect the mental health of children in mid-upper primary schools in Uganda.

The study innovates in several important ways. First, the interventions to be examined, family strengthening and economic empowerment, are to a great extent “home-grown” in SSA. There is robust evidence that shows that the economic empowerment intervention proposed in this study significantly improves the social circumstances of families in Uganda and has been associated with positive outcomes for youth, including some mental health outcomes (e.g., depression and hopelessness), particularly those made vulnerable by HIV/AIDS (18, 22, 23, 25, 74, 125). The family strengthening intervention has successfully improved the familial circumstances and the behavioral health of children in South Africa and Uganda (27, 28, 81, 170). To date, many approaches to child health and mental health in SSA communities have primarily been “transported” from outside the region, mainly from the global north (28, 34, 35, 73, 82, 186). We know little about how economic empowerment and family strengthening interventions might be deployed and scaled in combination and separately to improve child behavioral health. Moreover, gaps still exist in regard to the mechanisms by which economic empowerment and family strengthening interventions targeting social, familial, and context-specific drivers affect the mental health functioning of young people. Such knowledge is imperative as we begin to build and implement a child mental health policy agenda in LMICs. Therefore, it is critical to identify efficacious and potentially replicable intervention strategies developed and tested within the global south’s existing institutions and infrastructure and proven to have longer-term effects.

Second, existing child behavioral health interventions are largely tested in resource-rich settings with well-established health and social safety net systems that require considerable resources and staff time, thereby precluding wide dissemination in resource-constrained settings. Third, most existing child behavioral health interventions have failed to explicitly include economic empowerment components in child mental health treatment to address the well-documented structural economic factors. This failure constitutes a major gap, especially in the context of resource-constrained settings. Fourth, both economic empowerment and family strengthening have important additional strengths. They are theoretically driven; comprehensive, with a range of contextual and modifiable individual and social/psychosocial predictors; and designed to be able to engage children and families with serious and highly diverse needs and circumstances. Fifth, our study will be set in a region heavily affected by HIV/AIDS and other health epidemics, as well as a history of serious conflict. We may learn the extent to which participation in either or combined MFG-FS and EE may potentially change the would-be poor trajectory of mental health for children in low-resource communities.

Finally, the study design with a 2-year longitudinal follow-up allows for the study of successful and problematic CBH and family-level trajectories over a longer-term time horizon in order to inform strategic intervention points for children in one of the world’s poorest regions. The results may be relevant for others from impoverished backgrounds coping with chronic health conditions for which the literature on approaches and timing of interventions is limited.

## Conclusion

To date, we know little about the impact of a combined MFG-based family strengthening and economic empowerment intervention on child and adolescent mental health; potentially explaining the mixed and often non-sustained results of exclusively single interventions such as psychosocial counseling. Findings from this study can inform group, community, and population approaches that are needed for scalable solutions to address the social drivers negatively impacting child behavioral health in low-resource settings, including in SSA.

## Ethics statement

All study procedures were approved by the Washington University in St. Louis Review Board (IRB # 202202183) and by in-country local IRBs in Uganda: Uganda Virus Research Institute–UVRI (GC/127/901) and Uganda National Council of Science and Technology–UNCST (SS1205ES).

## Author contributions

FMS and MMM were the co-principal investigators for the grant. OSB, NN, SG, and PrN served as co-investigators. FMS, MMM, and OSB contributed to the conceptualization and methodology of the study. TN developed the statistical data analysis plan. NN, OSB, and PrN oversaw the implementation of the research study in collaboration with FMS and MMM. JK was the project coordinator of the study in the United States and PhN in Uganda. AM was the in-country implementation partner. FMS, MMM, OSB, JK, and PrN drafted the manuscript. All authors reviewed and commented on drafts and approved the final manuscript prior to submission.

## References

[1] Kieling C Baker-Henningham H Belfer M Conti G Ertem I Omigbodun O Child and adolescent mental health worldwide: evidence for action. *Lancet.* (2011) 378:1515–25. 22008427 10.1016/S0140-6736(11)60827-1

[2] Roberts M Mogan C Asare JB. An overview of Ghana’s mental health system: results from an assessment using the World Health Organization’s Assessment Instrument for Mental Health Systems (WHO-AIMS). *Int J Ment Health Syst.* (2014) 8:1–13. 10.1186/1752-4458-8-16 24817908 PMC4016652

[3] Cortina MA Sodha A Fazel M Ramchandani PG. Prevalence of child mental health problems in Sub-Saharan Africa: a systematic review. *Arch Pediatr Adolesc Med.* (2012) 166:276–81. 22393184 10.1001/archpediatrics.2011.592

[4] Belfer ML. Child and adolescent mental disorders: the magnitude of the problem across the globe. *J Child Psychol Psychiatry.* (2008) 49:226–36. 18221350 10.1111/j.1469-7610.2007.01855.x

[5] Burke JD Loeber R Birmaher B. Oppositional defiant disorder and conduct disorder: a review of the past 10 years, part II. *J Am Acad Child Adolesc Psychiatry.* (2002) 41:1275–93. 10.1097/00004583-200211000-00009 12410070

[6] Loeber R Burke JD Lahey BB Winters A Zera M. Oppositional defiant and conduct disorder: a review of the past 10 years, part I. *J Am Acad Child Adolesc Psychiatry.* (2000) 39:1468–84. 11128323 10.1097/00004583-200012000-00007

[7] Loeber R Green SM Lahey BB Frick PJ McBurnett K. Findings on disruptive behavior disorders from the first decade of the Developmental Trends Study. *Clin Child Fam Psychol Rev.* (2000) 3:37–60. 10.1023/a:1009567419190 11228766

[8] Patel V Flisher AJ Hetrick S McGorry P. Mental health of young people: a global public-health challenge. *Lancet.* (2007) 369:1302–13. 17434406 10.1016/S0140-6736(07)60368-7

[9] Patel V Rahman A. Editorial commentary: an agenda for global child mental health. *Child Adolesc Ment Health.* (2015) 20:3–4. 32680324 10.1111/camh.12083

[10] Vostanis P. Editorial: global child mental health – emerging challenges and opportunities. *Child Adolesc Ment Health.* (2017) 22:177–8. 10.1111/camh.12246 32680416

[11] Kohrt BA Asher L Bhardwaj A Fazel M Jordans MJD Mutamba BB The role of communities in mental health care in low- and middle-income countries: a meta-review of components and competencies. *Int J Environ Res Public Health.* (2018) 15:1279. 10.3390/ijerph15061279 29914185 PMC6025474

[12] World Health Organization. *Mental Health: Evidence and Research Department of Mental Health and Substance Dependence World Health Organization. Prevention and Promotion in Mental Health.* Geneva: World Health Organization (2002).

[13] Kaggwa EB Hindin MJ. The psychological effect of orphanhood in a matured HIV epidemic: an analysis of young people in Mukono, Uganda. *Soc Sci Med.* (2010) 70:1002–10. 10.1016/j.socscimed.2009.12.002 20106578

[14] Curley J Ssewamala F Han CK. Assets and educational outcomes: child development accounts (CDAs) for orphaned children in Uganda. *Child Youth Serv Rev.* (2010) 32:1585. 10.1016/j.childyouth.2009.07.016 PMC2976060 21076646

[15] Han CK Ssewamala FM Wang JSH. Family economic empowerment and mental health among AIDS-affected children living in AIDS-impacted communities: evidence from a randomised evaluation in southwestern Uganda. *J Epidemiol Community Health (1978).* (2013) 67:225–30. 10.1136/jech-2012-201601 23410851 PMC3578660

[16] Karimli L Sewamala FM. Do savings mediate changes in adolescents’ future orientation and health-related outcomes? Findings from randomized experiment in Uganda. *J Adolesc Health.* (2015) 57:425. 10.1016/j.jadohealth.2015.06.011 26271162 PMC4583807

[17] Karimli L Ssewamala FM Neilands TB Wells CR Bermudez LG. Poverty, economic strengthening, and mental health among AIDS orphaned children in Uganda: mediation model in a randomized clinical trial. *Soc Sci Med.* (2019) 228:17. 10.1016/j.socscimed.2019.03.003 30870668 PMC6502261

[18] Ssewamala FM Curley J. *School Attendance of Orphaned Children in Sub-Saharan Africa: The Role of Family Assets. PsycNET [Internet]. Social Development Issues: Alternative Approaches to Global Human Needs.* (2006). Available from: https://psycnet.apa.org/record/2007-19576-001 (accessed May 16, 2022).

[19] Ssewamala FM Neilands TB Waldfogel J Ismayilova L. The impact of a comprehensive microfinance intervention on depression levels of AIDS-orphaned children in Uganda. *J Adolesc Health.* (2012) 50:346–52. 10.1016/j.jadohealth.2011.08.008 22443837 PMC3314188

[20] Tozan Y Sun S Capasso A Wang JSH Neilands TB Bahar OS Evaluation of a savings-led family-based economic empowerment intervention for AIDS-affected adolescents in Uganda: a four-year follow-up on efficacy and cost-effectiveness. *PLoS One.* (2019) 14:e0226809. 10.1371/journal.pone.0226809 31891601 PMC6938344

[21] Wang JSH Ssewamala FM Neilands TB Bermudez LG Garfinkel I Waldfogel J Effects of financial incentives on saving outcomes and material well-being: evidence from a randomized controlled trial in Uganda. *J Policy Anal Manage.* (2018) 37:602. 10.1002/pam.22065 30122799 PMC6092028

[22] Ssewamala FM Sensoy Bahar O Tozan Y Nabunya P Mayo-Wilson LJ Kiyingi J A combination intervention addressing sexual risk-taking behaviors among vulnerable women in Uganda: study protocol for a cluster randomized clinical trial. *BMC Women’s Health.* (2019) 19:1–21. 10.1186/s12905-019-0807-1 31419968 PMC6697981

[23] Ssewamala FM Wang JSH Brathwaite R Sun S Mayo-Wilson LJ Neilands TB Impact of a family economic intervention (Bridges) on health functioning of adolescents orphaned by HIV/AIDS: a 5-Year (2012-2017) cluster randomized controlled trial in Uganda. *Am J Public Health.* (2021) 111:504–13. 10.2105/AJPH.2020.306044 33476237 PMC7893332

[24] Ssewamala FM Wang JSH Neilands TB Bermudez LG Garfinkel I Waldfogel J Cost-effectiveness of a savings-led economic empowerment intervention for AIDS-affected adolescents in Uganda: implications for scale-up in low resource communities. *J Adolesc Health.* (2018) 62(Suppl. 1):S29. 10.1016/j.jadohealth.2017.09.026 29273115 PMC5744872

[25] Ssewamala FM Ismayilova L McKay M Sperber E Bannon W Alicea S. Gender and the effects of an economic empowerment program on attitudes toward sexual risk-taking among AIDS-orphaned adolescent youth in Uganda. *J Adolesc Health.* (2010) 46:372. 10.1016/j.jadohealth.2009.08.010 20307827 PMC2844862

[26] Cavazos-Rehg P Byansi W Xu C Nabunya P Sensoy Bahar O Borodovsky J The impact of a family-based economic intervention on the mental health of HIV-infected adolescents in uganda: results from suubi + adherence. *J Adolesc Health.* (2021) 68:742–9. 10.1016/j.jadohealth.2020.07.022 32980245 PMC7987910

[27] Bhana A Mellins CA Petersen I Alicea S Myeza N Holst H The VUKA family program: piloting a family-based psychosocial intervention to promote health and mental health among HIV infected early adolescents in South Africa. *AIDS Care.* (2014) 26:1–11. 10.1080/09540121.2013.806770 23767772 PMC3838445

[28] Mellins CA Nestadt D Bhana A Petersen I Abrams EJ Alicea S Adapting evidence-based interventions to meet the needs of adolescents growing up with HIV in South Africa: the VUKA case example. *Global Social Welfare.* (2014) 1:97–110. 10.1007/s40609-014-0023-8 25984440 PMC4431642

[29] Penner F Sharp C Marais L Shohet C Givon D Boivin M. Community-based caregiver and family interventions to support the mental health of orphans and vulnerable children: review and future directions. *New Dir Child Adolesc Dev.* (2020) 171:77–105. 10.1002/cad.20352 32618434

[30] Calzada EJ Huang KY Anicama C Fernandez Y Brotman LM. Test of a cultural framework of parenting with Latino families of young children. *Cultur Divers Ethnic Minor Psychol.* (2012) 18:285–96. 22686147 10.1037/a0028694 PMC4405111

[31] Huang KY Abura G Theise R Nakigudde J. Parental depression and associations with parenting and children’s physical and mental health in a sub-saharan african setting. *Child Psychiatry Hum Dev.* (2017) 48:517–27. 10.1007/s10578-016-0679-7 27544380 PMC5318298

[32] Bradley RH Corwyn RF. Caring for children around the world: a view from HOME. *Int J Behav Dev.* (2005) 29:468–78.

[33] Brathwaite R Ssewamala FM Sensoy Bahar O McKay MM Neilands TB Namatovu P The longitudinal impact of an evidence-based multiple family group intervention (Amaka Amasanyufu) on oppositional defiant disorder and impaired functioning among children in Uganda: analysis of a cluster randomized trial from the SMART Africa-Uganda scale-up study (2016–2022). *J Child Psychol Psychiatry.* (2022) 63:1252–60. 10.1111/jcpp.13566 34989404 PMC9256858

[34] Ewart CK. Social action theory for a public health psychology. *Am Psychol.* (1991) 46:931–46. 1958012 10.1037//0003-066x.46.9.931

[35] Sherraden M Gilbert N. *Assets and the Poor: A New American Welfare Policy. Center for Social Development Research [Internet].* (2009). Available online at: https://openscholarship.wustl.edu/csd_research/12 (accessed May 16, 2022).

[36] Shobe M Page-Adams D. Assets, future orientation, and well-being: exploring and assets, future orientation, and well-being: exploring and extending sherraden’s framework. *J Sociol Soc Welfare.* (2001) 28:7.

[37] Bowen M. Family systems theory and society. In: Lorio JP McClenathan L editors. *Georgetown Family Symposia: Volume II (1973–1974).* Washington, DC: Georgetown Family Center (1977).

[38] World Health Organization. *Atlas: Child and Adolescent Mental Health Resources: Global Concerns: Implications for the Future [Internet].* Geneva: World Health Organization (2005).

[39] Giel R Harding TW. Psychiatric priorities in developing countries. *Br J Psychiatry.* (1976) 128:513–22. 1276559 10.1192/bjp.128.6.513

[40] Nalugya-Sserunjogi J Rukundo GZ Ovuga E Kiwuwa SM Musisi S Nakimuli-Mpungu E. Prevalence and factors associated with depression symptoms among school-going adolescents in Central Uganda. *Child Adolesc Psychiatry Ment Health.* (2016) 10:39. 10.1186/s13034-016-0133-4 27800012 PMC5081935

[41] Nalunga J. *Depression Amongst Secondary School Adolescents in Mukono District, Uganda.* Kampala: Makerere University (2004).

[42] Abolfotouh MA. Behaviour disorders among urban schoolboys in south-western Saudi Arabia. *East Mediter Health J.* (1997) 3:274–83.

[43] Akpan MU Ojinnaka NC Ekanem E. Behavioural problems among schoolchildren in Nigeria. *South Afr J Psychiatry.* (2010) 16:6. 10.1097/MD.0000000000022409 32991470 PMC7523811

[44] Ashenafi Y Kebede D Desta M Alem A. Prevalence of mental and behavioral disorders in children in Ethiopia. *East Afr Med J.* (2001) 78:308–11. 12002109

[45] Fleitlich-Bilyk B Goodman R. Prevalence of child and adolescent psychiatric disorders in southeast Brazil. *J Am Acad Child Adolesc Psychiatry.* (2004) 43:727–34. 15167089 10.1097/01.chi.0000120021.14101.ca

[46] Liang H Flisher AJ Chalton DO. Mental and physical health of out of school children in a South African township. *Eur Child Adolesc Psychiatry.* (2002) 11:257–60. 10.1007/s00787-002-0294-y 12541003

[47] Kivumbi A Byansi W Damulira C Namatovu P Mugisha J Sensoy Bahar O Prevalence of behavioral disorders and attention deficit/hyperactive disorder among school going children in Southwestern Uganda. *BMC Psychiatry.* (2019) 19:1–8. 10.1186/s12888-019-2069-8 30943981 PMC6446353

[48] Atwine B Cantor-Graae E Bajunirwe F. Psychological distress among AIDS orphans in rural Uganda. *Soc Sci Med.* (2005) 61:555–64. 10.1016/j.socscimed.2004.12.018 15899315

[49] Bhargava A. AIDS epidemic and the psychological well-being and school participation of Ethiopian orphans. *Psychol Health Med.* (2007) 10:263–75.

[50] Cluver L Gardner F. The psychological well-being of children orphaned by AIDS in Cape Town, South Africa. *Ann Gen Psychiatry.* (2006) 5:8. 10.1186/1744-859X-5-8 PMC1557503 16848910

[51] Cluver L Gardner F. The mental health of children orphaned by AIDS: a review of international and southern African research. *J Child Adolesc Ment Health.* (2007) 19:1–17. 10.2989/17280580709486631 25865319

[52] Cluver LD Orkin M Gardner F Boyes ME. Persisting mental health problems among AIDS-orphaned children in South Africa. *J Child Psychol Psychiatry.* (2012) 53:363–70. 10.1111/j.1469-7610.2011.02459.x 21883206

[53] Mueller J Alie C Jonas B Brown E Sherr L. A quasi-experimental evaluation of a community-based art therapy intervention exploring the psychosocial health of children affected by HIV in South Africa. *Trop Med Int Health.* (2011) 16:57–66. 10.1111/j.1365-3156.2010.02682.x 21073640

[54] Nyamukapa CA Gregson S Wambe M Mushore P Lopman B Mupambireyi Z Causes and consequences of psychological distress among orphans in eastern Zimbabwe. *AIDS Care.* (2010) 22:988–96. 10.1080/09540121003615061 20552465 PMC2924569

[55] World Health Organization. *Uganda Country Profile.* (2013). Available online at: https://www.who.int/countries/uga/ (accessed May 16, 2022).

[56] Bellis MA Lowey H Leckenby N Hughes K Harrison D. Adverse childhood experiences: retrospective study to determine their impact on adult health behaviours and health outcomes in a UK population. *J Public Health (Oxf).* (2014) 36:81–91. 10.1093/pubmed/fdt038 23587573

[57] Carlson CL. The child with oppositional defiant disorder and conduct disorders in the family. In: Quay HE Hogan AE editors. *Handbook of Disruptive Behavior Disorders.* New York: Plenun Press (1999) 337–52.

[58] Farrington DP. Development of offending and antisocial behaviour from childhood: key findings from the cambridge study in delinquent development | office of justice programs. *J Child Psychol Psychiatry.* (1995) 1995:929–64. 10.1111/j.1469-7610.1995.tb01342.x 7593403

[59] Hawkins JD Catalano RF Miller JY. Risk and protective factors for alcohol and other drug problems in adolescence and early adulthood: implications for substance abuse prevention. *Psychol Bull.* (1992) 112:64–105. 1529040 10.1037/0033-2909.112.1.64

[60] Kazdin A. *Conduct Disorders in Childhood and Adolescence. Conduct Disorders in Childhood and Adolescence [Internet].* (2014). Available online at: https://www.record/1995-97760-000 (accessed May 16, 2022).

[61] Ledingham JE. Children and adolescents with oppositional defiant disorder and conduct disorder in the community. *Handbook of Disruptive Behavior Disorders*. (Vol. 16), Boston, MA: Springer (1999) 353–70.

[62] Washburn J Teplin L Voss L Simon C Abram K McClelland G. Psychiatric disorders among detained youths: a comparison of youths processed in juvenile court and adult criminal court. *Psychiatr Serv.* (2008) 59:965. 10.1176/ps.2008.59.9.965 18757588 PMC2718561

[63] UNICEF. *State of the World’s Children 2015 Country Statistical Tables: Uganda Statistics.* New York, NY: UNICEF (2015).

[64] Brownstein JN Bone LR Dennison CR Hill MN Kim MT Levine DM. Community health workers as interventionists in the prevention and control of heart disease and stroke. *Am J Prev Med.* (2005) 29 (5 Suppl. 1):128–33. 10.1016/j.amepre.2005.07.024 16389138

[65] Koenig MA Lutalo T Zhao F Nalugoda F Wabwire-Mangen F Kiwanuka N Domestic violence in rural Uganda: evidence from a community-based study. *Bull World Health Organ.* (2003) 81:53–60. 12640477 PMC2572313

[66] Ovuga E Boardman J Wasserman D. The prevalence of depression in two districts of Uganda. *Soc Psychiatry Psychiatr Epidemiol.* (2005) 40:439–45. 16003593 10.1007/s00127-005-0915-0

[67] Naker D. *Violence Against Children The Voices of Ugandan Children and Adults [Internet].* (2005). Available online at: www.gypsykat.com (accessed March 20, 2022).

[68] Kleintjes S Lund C Flisher AJ. A situational analysis of child and adolescent mental health services in Ghana, Uganda, South Africa and Zambia. *Afr J Psychiatry (Johannesbg).* (2010) 13:132–9. 10.4314/ajpsy.v13i2.54360 20473475

[69] Baffoe M. Stigma, discrimination & marginalization: gateways to oppression of persons with disabilities in ghana, West Africa. *J Educ Soc Res.* (2013) 3:1578.

[70] Laugharne R Appiah-Poku J Laugharne J Shankar R. Attitudes toward psychiatry among final-year medical students in Kumasi, Ghana. *Acad Psychiatry.* (2009) 33:71–5. 10.1176/appi.ap.33.1.71 19349450

[71] Sorsdahl K Stein DJ Grimsrud A Seedat S Flisher AJ Williams DR Traditional healers in the treatment of common mental disorders in South Africa. *J Nerv Ment Dis.* (2009) 197:434–41. 19525744 10.1097/NMD.0b013e3181a61dbc PMC3233225

[72] Belsey MA Sherr L. The definition of true orphan prevalence: trends, contexts and implications for policies and programmes. *Vulner Children Youth Stud.* (2011) 6:185–200.

[73] Ssewamala FM Alicea S Bannon WM Ismayilova L. A novel economic intervention to reduce HIV risks among school-going AIDS orphans in Rural Uganda. *J Adolesc Health.* (2008) 42:102. 10.1016/j.jadohealth.2007.08.011 18155037 PMC2824559

[74] Ssewamala FM Han CK Neilands TB. Asset ownership and health and mental health functioning among AIDS-orphaned adolescents: findings from a randomized clinical trial in rural Uganda. *Soc Sci Med.* (2009) 69:191–8. 10.1016/j.socscimed.2009.05.019 19520472 PMC2819297

[75] Ssewamala FM Ismayilova L. Integrating children’s savings accounts in the care and support of orphaned adolescents in Rural Uganda. *Soc Serv Rev.* (2009) 83:453. 10.1086/605941 PMC2863345 20445763

[76] Ssewamala FM Karimli L Han CK Ismayilova L. Social capital, savings, and educational performance of orphaned adolescents in sub-Saharan Africa. *Child Youth Serv Rev.* (2010) 32:1704. 10.1016/j.childyouth.2010.07.013 20948971 PMC2952632

[77] Ssewamala FM Han CK Neilands TB Ismayilova L Sperber E. Effect of economic assets on sexual risk-taking intentions among orphaned adolescents in Uganda. *Am J Public Health.* (2010) 100:483. 10.2105/AJPH.2008.158840 20075323 PMC2820050

[78] Curley J Ssewamala FM Nabunya P Ilic V Keun HC. Child development accounts (CDAs): an asset-building strategy to empower girls in Uganda. *Int Soc Work.* (2016) 59:18–31. 10.1177/0020872813508569 26900173 PMC4758992

[79] Nabunya P Ssewamala FM. The effects of parental loss on the psychosocial wellbeing of AIDS-orphaned children living in AIDS-impacted communities: does gender matter? *Child Youth Serv Rev.* (2014) 43:131. 10.1016/j.childyouth.2014.05.011 25067869 PMC4107308

[80] Ssewamala FM Nabunya P Ilic V Mukasa MN Ddamulira C. Relationship between family economic resources, psychosocial well-being, and educational preferences of AIDS-orphaned children in southern uganda: baseline findings. *Glob Soc Welf.* (2015) 2:75–86. 10.1007/s40609-015-0027-z 26146601 PMC4486644

[81] Sensoy Bahar O Byansi W Kivumbi A Namatovu P Kiyingi J Ssewamala FM From “4Rs and 2Ss” to “Amaka Amasanyufu” (Happy Families): adapting a U.S.-based evidence-based intervention to the Uganda Context. *Fam Process.* (2020) 59:1928. 10.1111/famp.12525 32027763 PMC7416434

[82] McKay MMK Alicea S Elwyn L McClain ZRB Parker G Small LA The development and implementation of theory-driven programs capable of addressing poverty-impacted children’s health, mental health, and prevention needs: CHAMP and CHAMP+, evidence-informed, family-based interventions to address HIV risk and care. *J Clin Child Adolesc Psychol.* (2014) 43:428–41. 10.1080/15374416.2014.893519 24787707 PMC4215567

[83] McKay MM Gopalan G Franco L Dean-Assael K Chacko A Jackson JM A collaboratively designed child mental health service model: multiple family groups for urban children with conduct difficulties. *Res Soc Work Pract.* (2011) 21:664. 10.1177/1049731511406740 22194642 PMC3243310

[84] Farmer E Lipscombe J Moyers S. Foster carer strain and its impact on parenting and placement outcomes for adolescents. *Br J Soc Work.* (2005) 35:237–53.

[85] Cottrell D Boston P. Practitioner review: the effectiveness of systemic family therapy for children and adolescents. *J Child Psychol Psychiatry.* (2002) 43:573–86. 12120854 10.1111/1469-7610.00047

[86] Kilgore K Snyder J Lentz C. The contribution of parental discipline, parental monitoring, and school risk to early-onset conduct problems in African American boys and girls. *Dev Psychol.* (2000) 36:835–45. 10.1037//0012-1649.36.6.835 11081706

[87] Loeber R Farrington DP Stouthamer-Loeber M van Kammen WB. *Antisocial Behavior and Mental Health Problems: Explanatory Factors in Childhood and Adolescence. Antisocial Behavior and Mental Health Problems.* (1998). Available online at: https://www.taylorfrancis.com/books/mono/10.4324/9781410602930/antisocial-behavior-mental-health-problems-rolf-loeber-david-farrington-magda-stouthamer-loeber-welmoet-van-kammen (accessed May 16, 2022).

[88] Chorpita BF Becker KD Daleiden EL. Understanding the common elements of evidence-based practice: misconceptions and clinical examples. *J Am Acad Child Adolesc Psychiatry.* (2007) 46:647–52. 17450056 10.1097/chi.0b013e318033ff71

[89] Jones TL Prinz RJ. Potential roles of parental self-efficacy in parent and child adjustment: a review. *Clin Psychol Rev.* (2005) 25:341–63. 15792853 10.1016/j.cpr.2004.12.004

[90] Bruckner TA Scheffler RM Shen G Yoon J Chisholm D Morris J The mental health workforce gap in low- and middle-income countries: a needs-based approach. *Bull World Health Organ.* (2011) 89:184–94. 10.2471/BLT.10.082784 21379414 PMC3044251

[91] Kazdin AE Whitley MK. Treatment of parental stress to enhance therapeutic change among children referred for aggressive and antisocial behavior. *J Consult Clin Psychol.* (2003) 71:504–15. 12795574 10.1037/0022-006x.71.3.504

[92] Keiley MK. The Development and Implementation of an Affect Regulation and Attachment Intervention for Incarcerated Adolescents and their Parents. *Fam J.* (2002) 10:177–89.

[93] Kumpfer KL Alvarado R Smith P Bellamy N. Cultural sensitivity and adaptation in family-based prevention interventions. *Prev Sci.* (2002) 3:241–6. 12387558 10.1023/a:1019902902119

[94] Wahler RG Dumas JE. Attentional problems in dysfunctional mother-child interactions: an interbehavioral model. *Psychol Bull.* (1989) 105:116–30. 10.1037/0033-2909.105.1.116 2648437

[95] Webster-Stratton C Hammond M. Predictors of treatment outcome in parent training for families with conduct problem children. *Behav Ther.* (1990) 21:319–37.

[96] Sexton TL Alexander JF. Family-based empirically supported interventions. *Counsel Psychol.* (2016) 30:238–61.

[97] Carr A. Evidence-based practice in family therapy and systemic consultation. *J Fam Ther.* (2000) 22:29–60.

[98] Dishion TJ Patterson GR. The development and ecology of antisocial behavior in children and adolescents. *Dev Psychopathol.* (2006) 3:503–41.

[99] World Health Organization. *Mental Health Atlas.* Geneva: World Health Organization (2011).

[100] S.A.M.H.S.A. *The 4 Rs and 2 Ss for Strengthening Families Program.* (2015). Available online at: http://nrepp.samhsa.gov/ProgramProfile.aspx?id=41 (accessed February 1, 2017).

[101] Bhana A Petersen I Mason A Mahintsho Z Bell C McKay M. Children and youth at risk: adaptation and pilot study of the CHAMP (Amaqhawe) programme in South Africa. *Afr J AIDS Res.* (2004) 3:33–41. 10.2989/16085900409490316 25874981

[102] Nabunya P Ssewamala FM Ilic V. Family economic strengthening and parenting stress among caregivers of AIDS-orphaned children: results from a cluster randomized clinical Trial in Uganda. *Child Youth Serv Rev.* (2014) 44:417–21. 10.1016/j.childyouth.2014.07.018 25136142 PMC4133737

[103] Ismayilova L Ssewamala FM Karimli L. Family support as a mediator of change in sexual risk-taking attitudes among orphaned adolescents in rural Uganda. *J Adolesc Health.* (2012) 50:228–35. 10.1016/j.jadohealth.2011.06.008 22325127 PMC3279703

[104] Karimli L Ssewamala FM Neilands TB. Poor families striving to save in matched children’s savings accounts: findings from a randomized experimental design in Uganda. *Soc Serv Rev.* (2014) 88:658–94. 10.1086/679256 25525282 PMC4267259

[105] Arat G Wong PWC. The relationship between parental involvement and adolescent mental health in six sub-Saharan African countries: findings from Global School-based Health Surveys (GSHS). *Int J Mental Health Prom.* (2016) 18:144–57.

[106] Atilola O. Where lies the risk? An ecological approach to understanding child mental health risk and vulnerabilities in Sub-Saharan Africa. *Psychiatry J.* (2014) 2014:698348. 10.1155/2014/698348 24834431 PMC4009193

[107] Cluver L Boyes M Orkin M Sherr L. Poverty, AIDS and child health: identifying highest-risk children in South Africa. *South Afr Med J.* (2013) 103:910–5. 10.7196/samj.7045 24300627

[108] Cluver L Gardner F Operario D. Poverty and psychological health among AIDS-orphaned children in Cape Town. *South Africa. AIDS Care.* (2009) 21:732–41. 10.1080/09540120802511885 19806489

[109] Hasumi T Ahsan F Couper CM Aguayo JL Jacobsen KH. Parental involvement and mental well-being of Indian adolescents. *Indian Pediatr.* (2012) 49:915–8. 22728626 10.1007/s13312-012-0218-y

[110] McLeod JD Shanahan MJ. Poverty, parenting, and children’s mental health. *Am Sociol Rev.* (1993) 58:351–66.

[111] Reiss F. Socioeconomic inequalities and mental health problems in children and adolescents: a systematic review. *Soc Sci Med.* (2013) 90:24–31. 23746605 10.1016/j.socscimed.2013.04.026

[112] Peterson GW Rollins BC Thomas DL. Parental influence and adolescent conformity: compliance and internalization. *Youth Society.* (2016) 16:397–420.

[113] Mcneely C Shew ML Beuhring T Sieving R Miller BC Blum RWM. Mothers’ influence on the timing of first sex among 14- and 15-year-olds. *J Adolesc Health.* (2002) 31:256–65. 10.1016/s1054-139x(02)00350-6 12225738

[114] Miller-Johnson S Emery RE Marvin RS Clarke W Lovinger R Martin M. Parent-child relationships and the management of insulin-dependent diabetes mellitus. *J Consult Clin Psychol.* (1994) 62:603–10. 8063987 10.1037//0022-006x.62.3.603

[115] Resnick MD Bearman PS Blum RW Bauman KE Harris KM Jones J Protecting adolescents from harm. Findings from the national longitudinal study on adolescent health. *JAMA.* (1997) 278:823–32. 9293990 10.1001/jama.278.10.823

[116] Rohner RP. The parental “acceptance-rejection syndrome”: universal correlates of perceived rejection. *Am Psychol.* (2004) 59:830–40. 10.1037/0003-066X.59.8.830 15554863

[117] Rohner EC Rohner RP Roll S. Perceived parental acceptance-rejection and children’s reported behavioral dispositions: a comparative and intracultural study of american and mexican children. *J Cross Cult Psychol.* (2016) 11:213–31.

[118] Khaleque A Rohner RP. Perceived parental acceptance-rejection and psychological adjustment: a meta-analysis of crosscultural and intracultural studies. *J Marr Fam.* (2002) 64:54–64.

[119] Rohner RP Brothers SA. Perceived parental rejection, psychological maladjustment, and borderline personality disorder. *J Emot Abuse.* (1999) 1:81–95.

[120] Rohner RP Britner PA. Worldwide mental health correlates of parental acceptance-rejection: review of cross-cultural and intracultural evidence. *Cross Cult Res.* (2016) 36:16–47.

[121] Amerikaner M. Family interaction and individual psychological health. *J Counsel Dev.* (1994) 72:614.

[122] Sherraden M. Stakeholding: notes on a theory of welfare based on assets. *Soc Serv Rev.* (2015) 64:580–601.

[123] Garmezy N. Stress-resistant children: the search for protective factors. In: Stevenson J editor. *Recent Research in Developmental Psychopathology: Journal of Child Psychology and Psychiatry Book Supplement.* Oxford: Pergamon (1985) 213–33.

[124] Garmezy N. Reflections and commentarty on risk, resilience and development. In: Haggarty R editor. *Stress, Risk and Reslilience in Children and Adolescents: Processes, Mechanisms and Interventions.* New York, NY: Cambridge University Press (1994).

[125] Ssewamala FM Sperber E Zimmerman JM Karimli L. The potential of asset-based development strategies for poverty alleviation in Sub-Saharan Africa. *Int J Soc Welf.* (2010) 19:433–43.

[126] Zhan M Sherraden M. Assets, expectations, and children’s educational achievement in female-headed households. *Soc Serv Rev.* (2003) 77:191–211.

[127] Rutherford S. *The Poor and Their Money.* New Delhi: Oxford University Press (2000).

[128] Bandura A. Social cognitive theory: an agentic perspective. *Annu Rev Psychol.* (2003) 52:1–26. 10.1146/annurev.psych.52.1.1 11148297

[129] Ajzen I. The theory of planned behavior. *Organiz Behav Hum Decis Proces.* (1991) 50:179–211.

[130] Ajzen I Fishbein M. *Understanding Attitudes and Predicting Social Behavior.* Hoboken, NJ: Prentice-Hall (1980). 278 p.

[131] Fishbein M. *Belief, Attitude, Intention, and Behavior: An Introduction to Theory and Research BibSonomy.* Reading, MA: Addison-Wesley (1975).

[132] Cochran SD Mays VM. Applying social psychological models to predicting HIV-related sexual risk behaviors among african americans. *J Black Psychol.* (1993) 19:142. 10.1177/00957984930192005 23529205 PMC3606488

[133] Fischbein M. Attitude and prediction of behavior. In: Fischbein M editor. *Readings in Attitude Theory and Measurement.* New York, NY: John Wiley (1967) 477–92.

[134] Jemmott JB Jemmott LS Hacker CI. Predicting intentions to use condoms among African-American adolescents: the theory of planned behavior as a model of HIV risk-associated behavior. *Ethn Dis.* (1992) 2:371–80. 1490134

[135] de Walque D Dow WH Nathan R Abdul R Abilahi F Gong E Incentivising safe sex: a randomised trial of conditional cash transfers for HIV and sexually transmitted infection prevention in rural Tanzania. *BMJ Open.* (2012) 2:e000747. 10.1136/bmjopen-2011-000747 22318666 PMC3330254

[136] Baird SJ Garfein RS McIntosh CT Özler B. Effect of a cash transfer programme for schooling on prevalence of HIV and herpes simplex type 2 in Malawi: a cluster randomised trial. *Lancet.* (2012) 379:1320–9. 10.1016/S0140-6736(11)61709-1 22341825

[137] Cluver L Boyes M Orkin M Pantelic M Molwena T Sherr L. Child-focused state cash transfers and adolescent risk of HIV infection in South Africa: a propensity-score-matched case-control study. *Lancet Glob Health.* (2013) 1:e362–70. 10.1016/S2214-109X(13)70115-3 25104601

[138] Ranganathan M Lagarde M. Promoting healthy behaviours and improving health outcomes in low and middle income countries: a review of the impact of conditional cash transfer programmes. *Prev Med.* (2012) 55:S95–105. 10.1016/j.ypmed.2011.11.015 22178043

[139] Pettifor A MacPhail C Nguyen N Rosenberg M. Can money prevent the spread of HIV? A review of cash payments for HIV prevention. *AIDS Behav.* (2012) 16:1729–38. 10.1007/s10461-012-0240-z 22760738 PMC3608680

[140] Kagotho N Ssewamala FM. Correlates of depression among caregivers of children affected by HIV/AIDS in Uganda: findings from the suubi-maka family study. *AIDS Care.* (2012) 24:1226. 10.1080/09540121.2012.658754 PMC3422693 22375826

[141] Uganda Aids Commission Secretariat. *Factsheet- Facts on HIV and AIDS in Uganda 2021 (Based on Data ending 31st december 2020).* (2021). Available online at: https://uac.go.ug/media/attachments/2021/09/13/final-2021-hiv-aids-factsheet.pdf (accessed March 20, 2022).

[142] Hayes RJ Moulton LH. *Choice of Analytical Method Cluster Randomised Trials.* London: Chapman & Hall/CRC (2009) 223–4.

[143] Jennings L Ssewamala FM Nabunya P. Effect of savings-led economic empowerment on HIV preventive practices among orphaned adolescents in rural Uganda: results from the Suubi-Maka randomized experiment. *AIDS Care.* (2016) 28:273. 10.1080/09540121.2015.1109585 26548549 PMC4747687

[144] Ssewamala FM Curley JC. Attendance of orphaned children in sub-saharan africa: the role of family assets. *Soc Dev Issues Altern Approac Global Hum Needs.* (2006) 28:84–105.

[145] Ssewamala FM Karimli L Torsten N Wang JSH Han CK Ilic V Applying a family-level economic strengthening intervention to improve education and health-related outcomes of school-going AIDS-orphaned children: lessons from a randomized experiment in Southern Uganda. *Prev Sci.* (2016) 17:134–43. 10.1007/s11121-015-0580-9 26228480 PMC4697878

[146] Dennison ST. *A Multiple Family Group Therapy Program for at Risk Adolescents and Their Families.* Springfield, IL: Charles C Thomas Publisher (2005). 310 p.

[147] McKay MM Gonzales JJ Stone S Ryland D Kohner K. Multiple family therapy groups: a responsive intervention model for inner City Families. *Soc Work Groups.* (1996) 18:41–56.

[148] Karimli L Ssewamala FM Neilands TB McKay MMK. Matched child savings accounts in low-resource communities: who saves? *Global Social Welfare.* (2015) 2:53–64. 10.1007/s40609-015-0026-0 26636025 PMC4664459

[149] O’SHEA MD Phelps R. Multiple family therapy: current status and critical appraisal. *Fam Process.* (1985) 24:555–82. 3910450 10.1111/j.1545-5300.1985.00555.x

[150] Jewell TC Downing D McFarlane WR. Partnering with families: multiple family group psychoeducation for schizophrenia. *J Clin Psychol.* (2009) 65:868–78. 10.1002/jclp.20610 19530233

[151] Chacko A Gopalan G Franco L Dean-Assael K Jackson J Marcus S Multiple family group service model for children with disruptive behavior disorders: child outcomes at post-treatment. *J Emot Behav Disord.* (2015) 23:67. 10.1177/1063426614532690 PMC4548959 26316681

[152] McKay M Block M Mellins C Traube DE Brackis-Cott E Minott D Adapting a family-based HIV prevention program for HIV-infected preadolescents and their families: youth, families and health care providers coming together to address complex needs. *Soc Work Ment Health.* (2007) 5:355. 10.1300/J200v05n03_06 20852676 PMC2939450

[153] Ssewamala FM Nabunya P Mukasa NM Ilic V Nattabi J. Integrating A mentorship component in programming for care and support of AIDS-orphaned and vulnerable children: lessons from the suubi and bridges programs in sub-Saharan Africa. *Glob Soc Welf.* (2014) 1:9. 10.1007/s40609-014-0008-7 24999449 PMC4078881

[154] Ssewamala FM Bermudez LG Neilands TB Mellins CA McKay MM Garfinkel I Suubi4Her: a study protocol to examine the impact and cost associated with a combination intervention to prevent HIV risk behavior and improve mental health functioning among adolescent girls in Uganda. *BMC Public Health.* (2018) 18:45879. 10.1186/s12889-018-5604-5 29871619 PMC5989412

[155] Beck AT Weissman A Lester D Trexler L. The measurement of pessimism: the hopelessness scale. *J Consult Clin Psychol.* (1974) 42:861–5. 4436473 10.1037/h0037562

[156] Frederick C Pynoos R Nader K. *Childhood Post-Traumatic Stress Reaction Index (CPTS-RI) [Copyrighted Semi-Structured Interview].* Los Angeles, CA: Two Suns Measures (1992).

[157] Fitts WH Warren WL. *Tennessee Self-Concept Scale: Tscs-2. Manual.* 2nd Edn. Los Angeles, CA: Western Psychological Services (1996).

[158] Tolan PH Gorman-Smith D Huesmann LR Zelli A. Assessment of family relationship characteristics: a measure to explain risk for antisocial behavior and depression among Urban. *Psychol Asses.* (1997) 9:212–23.

[159] Karimli L Ssewamala FM Ismayilova L. Extended families and perceived caregiver support to AIDS orphans in Rakai district of Uganda. *Child Youth Serv Rev.* (2012) 34:1351–8. 10.1016/j.childyouth.2012.03.015 23188930 PMC3505487

[160] Hamilton E Carr A. Systematic review of self-report family assessment measures. *Fam Process.* (2016) 55:16–30. 26582601 10.1111/famp.12200

[161] Vaux A Riedel S Stewart D. Modes of social support: the social support behaviors (SS-B) scale. *Am J Commun Psychol.* (1987) 15:209–32.

[162] Zimet GD Dahlem NW Zimet SG Farley GK. The multidimensional scale of perceived social support. *J Pers Assess.* (1988) 52:30–41. 10.1080/00223891.1990.9674095 2280326

[163] Kaushik A Papachristou E Dima D Fewings S Kostaki E Ploubidis GB Measuring stigma in children receiving mental health treatment: validation of the Paediatric Self-Stigmatization Scale (PaedS). *Eur Psychiatry.* (2017) 43:1–8. 10.1016/j.eurpsy.2017.01.004 28371742

[164] Rosenberg M. Rosenberg Self-Esteem Scale (RSE). Acceptance and Commitment Therapy Measures Package. (1965). Available online at: https://integrativehealthpartners.org/downloads/ACTmeasures.pdf#page=61 (accessed March 10, 2022).

[165] Coates J Swindale A Bilinsky P. *Household Food Insecurity Access Scale (HFIAS) for Measurement of Food Access: Indicator Guide*. Washington, DC: Food and Nutrition Technical Assistance (2007)

[166] Bermudez LG Jennings L Ssewamala FM Nabunya P Mellins C McKay M. Equity in adherence to antiretroviral therapy among economically vulnerable adolescents living with HIV in Uganda. *AIDS Care.* (2016) 28:83–91. 10.1080/09540121.2016.1176681 27392003 PMC4940111

[167] Microfinanceopportunities. *Financial Education Core Curriculum.* (2002). Available online at: https://www.microfinanceopportunities.org/4-work-with-us/mfo-in-the-field/project-list/fecc/ (accessed March 20, 2022).

[168] National Institute on Drug Abuse [NIDA]. *Risk Behavior Assessment.* 3rd Edn. Bethesda, MD: National Institute on Drug Abuse (1993).

[169] Waschbusch DA Willoughby MT. Parent and teacher ratings on the IOWA Conners Rating Scale. *J Psychopathol Behav Assess.* (2008) 30:180–92.

[170] Ssewamala FM Sensoy Bahar O McKay MM Hoagwood K Huang KY Pringle B. Strengthening mental health and research training in Sub-Saharan Africa (SMART Africa): Uganda study protocol. *Trials.* (2018) 19:423. 10.1186/s13063-018-2751-z 30081967 PMC6080393

[171] Palinkas LA Horwitz SM Green CA Wisdom JP Duan N Hoagwood K. Purposeful sampling for qualitative data collection and analysis in mixed method implementation research. *Adm Policy Ment Health.* (2015) 42:533. 10.1007/s10488-013-0528-y PMC4012002 24193818

[172] Lincoln YS Guba EG. *Naturalistic Inquiry.* Newbury Park, CA: Sage Publications (1985).

[173] Miles MB Huberman AM Huberman MA Huberman M. *Qualitative Data Analysis: An Expanded Sourcebook.* Thousand Oaks, CA: Sage (1994).

[174] Strauss A Corbin J. *Basics of Qualitative Research: Techniques and Procedures for Developing Grounded Theory.* 2nd Edn. Thousand Oaks, CA: Sage publications (1998). 333 p.

[175] Center for Social Development. *MIS IDA Operations Manual: Management Information System for Individual Development Accounts.* St. Louis, MI: Washington University (2022).

[176] Muthén B Asparouhov T. Causal effects in mediation modeling: an introduction with applications to latent variables. *Struct Equat Model.* (2014) 22:12–23. 10.1007/s00038-010-0198-4 20931349

[177] Power Analysis Software. *Sample Size Software PASS [Internet].* (2022). Available online at: https://www.ncss.com/software/pass/ (accessed May 16, 2022).

[178] Kahana SY Rohan J Allison S Frazier TW Drotar D. A meta-analysis of adherence to antiretroviral therapy and virologic responses in HIV-infected children, adolescents, and young adults. *AIDS Behav.* (2013) 17:41–60. 10.1007/s10461-012-0159-4 22411426

[179] QSR International Pty Ltd. *NVivo* (2020). Available online at: https://www.qsrinternational.com/nvivo-qualitative-data-analysis-software/home (accessed March 2020).

[180] Charmaz K. Grounded theory: objectivist and constructivist methods. In: Denzin NK Lincoln YS editors. *Strategies for Qualitative Inquiry.* Thousand Oaks, CA: Sage (2003) 249–91. 10.1016/j.nedt.2006.09.001

[181] Boyatzis RE. *Transforming Qualitative Information: Thematic Analysis and Code Development.* Thousand Oaks, CA: Sage (1998).

[182] Padgett DK. *Qualitative Methods in Social Work Research.* Thousand Oaks, CA: SAGE Sourcebooks for the Human Services (2017). 352.

[183] Creswell JW Clark VLP. *Designing and Conducting Mixed Methods Research + the Mixed Methods Reader*, Vol. 1. Cincinnati, OH: University of Cincinnati (2017) 24–7.

[184] Greene JC Caracelli VJ. *Advances in Mixed-Method Evaluation: The Challenges and Benefits of Integrating Diverse Paradigms.* San Francisco, CA: Jossey-Bass Publishers (1997). 97.

[185] Greene JC Caracelli VJ Graham WF. Toward a conceptual framework for mixed-method evaluation designs. *Educ Evaluat Policy Analys.* (2016) 11:255–74. 10.3102/01623737011003255

[186] Schreiner M Clancy M Sherraden M Warren G. *Saving Performance in the American Dream Demonstration: A National Demonstration of Individual Development Accounts. Center for Social Development Research [Internet].* (2002). Available online at: https://openscholarship.wustl.edu/csd_research/343 (accessed May 16, 2022).

